# Polyneuropathy in Parkinson’s disease and atypical Parkinsonian syndromes: clinical impact and risk factors

**DOI:** 10.1007/s00702-025-03032-9

**Published:** 2025-10-09

**Authors:** Amélie L. Conring, Lan Ye, Florian Wegner, Sonja Körner, Tobias Hegelmaier, Matthias Höllerhage, Martin Klietz

**Affiliations:** Department of Neurology, Hannover Medical School, Carl-Neuberg-Straße 1, 30625 Hannover, Germany

**Keywords:** Parkinson’s disease, Progressive supranuclear palsy, Multiple system atrophy, Polyneuropathy

## Abstract

Polyneuropathy (PNP) is increasingly recognized as a comorbidity in Parkinson’s disease (PD) but its prevalence, clinical features and impact in atypical Parkinsonian syndromes (APS) remain unclear. Understanding PNP in Parkinsonism is crucial for improving patients’ mobility, slowing the disability progression and reducing disease burden. This study aims to characterize prevalence, etiology, and clinical relevance of PNP in PD and APS. Consecutive admissions of 104 PD, 52 progressive supranuclear palsy (PSP), and 27 multiple system atrophy (MSA) patients to the Department of Neurology at Hannover Medical School were analyzed. Assessments included Hoehn and Yahr stage, MDS-UPDRS III, electroneurography, PNP related conditions (e.g., diabetes mellitus, vitamin deficiencies), and PD drugs including levodopa equivalence dose (LED). PNP was highly prevalent across all three groups with a prevalence ranging from 37.0% and 47.1%. PD and PSP patients with PNP were older and predominantly male (p < 0.05). They also showed more advanced Hoehn and Yahr stages and higher MDS-UPDRS-III scores (p < 0.05). Sensorimotor axonal, length‐dependent peripheral neuropathy was the most frequent presentation. We found no association between PNP and PD medications, and classic risk factors for PNP, such as diabetes mellitus, vitamin B12 deficiency, or chronic alcohol abuse, did not differ significantly between patients with and without PNP. Screening for PNP among patients with Parkinsonism, particularly among older males, is critical to optimize mobility. Further studies to identify the cause of the high prevalence of PNP among this population are needed.

## Introduction

Polyneuropathy (PNP) is a peripheral neurological disorder, clinically characterized by symmetrical distal numbness, paresthesia and often accompanied by pain and weakness, leading to impaired balance and gait disturbance that can significantly impact daily functioning and mobility (Sommer et al. 2018). Up to 7% of adults are affected by PNP (Elafros et al. 2022) though the prevalence of PNP is reported to be even higher in patients with Parkinson's disease (PD) than in the general population (Toth et al. 2010; Merola et al. 2017; Corrà et al. 2023).

PD is the second most common neurodegenerative disease and the leading cause of Parkinsonism (Aarsland et al. 2021). It manifests clinically with multiple motor symptoms such as akinesia, rigidity, tremor at rest, and postural instability. Gait disturbance and postural instability can be aggravated by PNP (Corrà et al. 2023). Atypical Parkinsonian syndromes (APS), such as progressive supranuclear palsy (PSP) and multiple system atrophy (MSA), share overlapping clinical features with PD but progress more rapidly, leading to a more severe functional decline (Fabbrini et al. 2019). Patients with PD and APS present a specific profile of comorbidities (Ye et al. 2024; Greten et al. 2024; Klietz et al. 2019). Previous findings suggested that PNP might have a high prevalence and impact on disease burden in APS patients (Rohmann et al. 2021), however, the exact connections remain unknown.

Despite the intensive investigation of the relationship between PNP and PD, the exact linkage between the two is still unclear. In the general population, the common causes of PNP are diabetes mellitus type 2 (T2DM), elevated alcohol consumption, or vitamin deficiencies (Hanewinckel et al. 2016a). While previous studies have shown that T2DM is closely associated with PD (Stockmann et al. 2025), it is important to investigate whether T2DM is also the primary cause of PNP in the PD population. Additionally, it has been hypothesized that levodopa, which is the most common medical treatment in PD patients, may play a causative role in the development of PNP in PD (Romagnolo et al. 2019), whereas catechol-O-methyltransferase (COMT) inhibitors, another commonly used class of PD drugs, might prevent this development (Cossu et al. 2016; Müller 2011). It is crucial to investigate this hypothesis in a larger clinical setting to ensure safe and efficient medical support for patients. Moreover, given the different pathogeneses among different Parkinsonism diseases, it is crucial to determine whether the risk factors for PNP in PD are the same as for PNP in APS.

In this study, the prevalence, clinical and electrophysiological features, and risk factors of PNP in patients with PD, PSP, and MSA were systematically evaluated. The aim of this study was to determine whether shared or distinct mechanisms contribute to PNP development in these patients.

## Methods

### Participants

Ethical approval was obtained from the local Ethics Committee at Hannover Medical School (No. 8666_Bo_K_2019). Written informed consent was obtained from all patients in order to participate in this study. Cross-sectional data was collected from PD and PSP/MSA patients consecutively admitted to the Department of Neurology at Hannover Medical School between 02/2020 and 12/2024. The PD, PSP, and MSA diagnoses were made by a movement disorder specialist according to Movement Disorders Society diagnostic criteria for PD (Postuma et al. 2015), PSP (Höglinger et al. 2017), and MSA (Gilman et al. 2008). All patients diagnosed with PD, PSP, or MSA underwent a PNP screening. PNP was diagnosed according to the guideline by the German Neurological Society (DNG) (Heuß, et al. 2024). In total, 104 PD patients, 52 PSP patients, and 27 MSA patients were analyzed. Of those patients, 84 PD, 40 PSP, and 22 MSA patients received a complete PNP work up with targeted electrophysiological and laboratory assessments.

### Clinical evaluations

The obtained clinical data included sex, age at examination, age at onset of movement disorder, duration of disease, Hoehn and Yahr stage (Hoehn and Yahr 1967), Movement Disorder Society Unified Parkinson's Disease Rating Scale part III (MDS-UPDRS III) (Goetz et al. 2008), Montreal Cognitive Assessment (MoCA) to evaluate the global cognitive status (Nasreddine et al. 2005), and, for PD patients specifically, the Non-Motor Symptoms Scale (Chaudhuri et al. 2020). Additionally, symptoms or signs commonly related with neuropathy, including reduced reflexes, paresis, Romberg sign, ataxia, hypesthesia, pallhypesthesia, and gait disturbance (Sommer et al. 2018) were documented. Past medical history such as malignancy, vitamin B12 deficiency, diabetes, and the treatment records for PD-drugs were reviewed. The dosage of COMT inhibitors and the total levodopa equivalent daily doses (LEDD) were calculated according to current guidelines of the German Society of Neurology (Höllerhage et al. 2024).

### Electrophysiological assessment

NCS were performed following the established protocol (Filter setting: 5-10 Hz (motoric nerve), 20 Hz-1.5 kHz (sensoric nerve); weep speed: 5 ms/div (motoric nerve:), 1 ms/div (sensoric nerve); stimulation parameters: supermaximal between 40-100 mA (motoric nerve), supermaximal between 12 and 40 mA (sensoric nerve) for PNP in the Department of Neurology using superficial stimulators and recording electrodes as previously reported (Seeliger et al. 2021, 2019). The assessment included the ulnar nerve, median nerve, sural nerve, tibial nerve, and peroneal nerve on at least one side. If only one side was measured, the clinically more symptomatic side was chosen. The number of the measured nerves was in some cases tailored to the specific symptoms and their localization. The diagnosis of PNP, including its type (axonal, demyelinating, or mixed), quality (sensory, motor, or sensorimotor) and the distribution of the damage, was determined by our electrophysiological expert according to the criteria of the German Neurological Society (Heuß, et al. 2024). Axonal PNP is characterized by a uniform reduction in the amplitudes of muscle action potential (cMAP) upon distal and proximal stimulation, or a reduction in sensory nerve action potential (sNAP). Optionally, a reduction in nerve conduction velocity of up to 30% below the age-adjusted lower normal values may also be noted. Demyelinating PNP is characterized by a prolonged distal latency, decreased nerve conduction velocity, reduced amplitude of cMAP and prolonged cMAP duration at proximal stimulation resulting in pathological temporal dispersion, prolonged corrected F-wave latency and increased chrono dispersion. In some cases, demyelinating damage may also be associated with multiple A-waves in acute inflammatory demyelinating polyneuropathy. In the absence of standardized guidelines to determine the severity of polyneuropathy, we adopted the established approach described by Kwon et al. (Kwon et al. 2024), with slight modifications based on the reference standards used in our department (Bischoff and Dengler 2018). Briefly, among the patients with diagnosed PNP, the severity of PNP was further divided into mild, moderate, or severe. Mild PNP was defined as a sNAP below 4 µV. Moderate PNP was defined as an additional reduction of cMAP of the tibial nerve below 4 mV. Severe PNP was classified if, in addition to the above reductions, a further decrease was observed with a median nerve cMAP of less than 7 mV or a median sNAP of less than 5 µV after excluding carpal tunnel symptoms.

### Clinical laboratory measures

The standard laboratory tests included assessments of thyroid function (TSH), renal function (eGFR, Creatinine) and liver function (ALT, AST, GGT, Bilirubin), diabetes mellitus (blood glucose levels, HbA1c) as well as blood cell counts, vitamin B12 levels (incl. Vitamin B12 levels, Holotranscobalamin levels, Methylmalonic acid levels), Thiamine level, folic acid level, and immunofixation. For patients with a history of malignancy, anti-neuronal antibodies were tested. If applicable, cell count, leucocyte distribution, and protein levels in the cerebrospinal fluid (CSF) were analyzed. A lumbar puncture was only performed on patients where examination of the CSF was necessary for differential diagnosis and who had provided informed consent for the procedure.

### Statistical analysis

For the statistical analysis R studio Ver. 2024.09.1 + 394 by © 2025 Posit Software, PBC was used. Independent t-tests were used to compare normally distributed variables between two groups, while Mann–Whitney U tests were used to compare variables that did not meet the criteria of a normal distribution. One-way ANOVA followed by the Tukey post-hoc test was used to compare normally distributed variables among more than two groups, while Kruskal–Wallis-test followed by pairwise Wilcoxon test was used for not normally distributed data. The chi-square test was used to compare proportions for categorical variables.

## Results

### Comparison of PD, PSP, and MSA patients

A total of 104 PD patients, 52 PSP, and 27 MSA patients were analyzed. The average age at examination was 67.9 ± 10.7 years for PD, 69.5 ± 7.9 years for PSP, and 62.3 ± 8.6 years for MSA. The patients in the MSA group were significantly younger than the patients in the other two groups (MSA/PD: p = 0.016, MSA/PSP: p = 0.0021). Disease duration varied significantly across groups (p = 3.7e-7) and in all pairwise comparisons (MSA/PD: p = 0.032; MSA/PSP: p = 0.0069; PD/PSP: p = 6.5e-7), with mean durations of 7.3 ± 6.6 years for PD, 2.4 ± 1.7 years for PSP, and 3.6 ± 2.3 years for MSA. PSP patients received significantly lower doses of COMT inhibitors, levodopa daily doses (LDD), and LEDD compared to PD patients, with no significant differences compared to MSA patients (PSP vs. PD: COMT inhibitors p = 0.008; LDD p = 0.0026; LEDD p = 0.00011). Among the patient groups, 49 PD (47.1%), 23 PSP (44.2%), and 10 MSA patients (37.0%) were electrophysiologically diagnosed with polyneuropathy. There was no significant difference concerning the prevalence of polyneuropathy among all three disease groups (p = 0.641) (Table 1).

**Table 1:** Demographic characteristics in PD, PSP and MSA patients

	PD (n = 104)	PSP (n = 52)	MSA (n = 27)
Age (years)	*67.9* ± *10.7*	*69.5* ± *7.9*^*§*^	*62.3* ± *8.0*^*§#*^
Sex	*F 44.2% (46/104)* *M 55.8% (58/104)*	*F 44.2% (23/52)* *M 55.8% (29/52)*	*F 59.3% (16/27)* *M 40.7% (11/27)*
Duration (years)	*7.3* ± 6.6*	*2.4* ± *1.7*	*3.6* ± *2.3*
HY	*3.0* ± *1.0*	*3.0* ± *1.0*^*#*^	*3.5* ± *1.0*^*#*^
MDS-UPDRS III	*27.0* ± *15.0*	*35.0* ± *16.0*^*#*^	*44.0* ± *14.0*^*#*^
MoCA	*23.0* ± *4.0*	*22.5* ± *5.0*	*25* ± *3.0*
COMT (mg)	*41.2* ± *108.2*	*0.48* ± *3.5*^*#*^	*8.26* ± *38.2*
LDD (mg)	*410.1* ± *444.8*	*195.2* ± *270.5*^*#*^	*367.6* ± *437.8*
LEDD (mg)	*540.5* ± *518.9*	*223.4* ± *301.2*^*#*^	*439.3* ± *526.2*
Prevalence of PNP (%)	*47.1*	*44.2*	*35.7*

Data are presented as mean ± SD

*COMT* catechol-O-methyltransferase inhibitor, *HY* Hoehn and Yahr Stage, *MoCA* Montreal Cognitive Assessment, *MDS-UPDRS III* Movement Disorder Society Unified Parkinson’s Disease Rating Scale Part III, *MSA* multiple-system atrophy, *LDD* Levodopa Daily Dose, *LEDD* Levodopa equivalent daily dose, *PD* Parkinson’s Disease, *PNP* Polyneuropathy, *PSP* Progressive supranuclear palsy

^*^p < 0.05 among all three groups

^#^p < 0.05 PSP or MSA compared with PD

^§^p < 0.05 between PSP and MSA patients

### Comparison of patients with and without PNP in PD, PSP, and MSA

As shown in Table 2, among PD patients, significantly more men were suffering from PNP than women (p = 0.027). PD patients with PNP were significantly older than those without (72.4 ± 8.2 vs 63.9 ± 11.1 years old, p = 7.94e-5). Their motor deficits were also significantly more severe, which were measured by Hoehn and Yahr stage (p = 0.0088) and MDS-UPDRS III (p = 0.0087), although there was no significant difference in the duration of disease (p = 0.161) or in cognitive function, as measured by the MoCA (p = 0.276). The autonomic part of the MDS-NMSS, including urinary retention, orthostatic syndrome, sexual dysfunction, gastrointestinal tract symptoms, did not show differences in PD patients with and without PNP (data not shown). There was a trend towards PD patients with PNP receiving higher doses of COMT inhibitors, but the difference was not statistically significant (p = 0.176), even though PD patients with PNP had more advanced disease stages. No differences on either LDD or LEDD were observed in this study (LDD: p = 0.193, LEDD: p = 0.087).

**Table 2:** Clinical and biochemical characteristics in PD, PSP and MSA patients with and without PNP

	PD + PNP (n = 49)	PD-PNP (n = 55)	PSP + PNP (n = 23)	PSP-PNP (n = 29)	MSA + PNP (n = 10)	MSA-PNP (n = 17)
Age (yrs)	*72.4* ± 8.2*	*63.9* ± *11.1*	*72.7* ± 7.5*	*66.9* ± *7.5*	*61.8* ± *10.6*	*62.6* ± *7.43*
Sex	F 32.6% (16/49) * *M 67.3% (33/49)*	*F 54.5% (30/55)* *M 45.5% (25/55)*	F 26.1% (6/23) * *M 73.9% (17/23)*	*F 60.7% (17/29)* *M 39.3% (12/29)*	*F 40.0% (4/10)* *M 60.0% (6/10)*	*F 70.6% (12/17)* *M 29.4% (5/17)*
Duration (yrs)	*8.0* ± *6.4*	*6.6* ± *6.7*	*2.7* ± *1.8*	*2.2* ± *1.6*	*3.6* ± *2.8*	*3.7* ± *2.6*
HY	*3.0* ± 1.0*	*2.5* ± *1.0*	*4.0* ± 1.0*	*3.0* ± *1.0*	*4.0* ± *1.0*	*3.0* ± *1.0*
MDS-UPDRS III	*31.0* ± 15.0*	*23.0* ± *13.0*	*41.0* ± 18.0*	*30.0* ± *14.0*	*44.0* ± *19.0*	*43.0* ± *10.0*
MoCA	*22.0* ± *4.0*	*24.0* ± *4.0*	*21.0* ± *5.0*	*23.0* ± *4.0*	*26.0* ± *2.5*	*24.0* ± *3.5*
COMT (mg)	*59.6* ± *128.1*	*24.7* ± *84.6*	*1.1* ± *5.2*	*0.0*	*22.3* ± *62.2*	*0.0*
LDD (mg)	*451.0* ± *461.0*	*373.7* ± *430.9*	*251.1* ± *284.1*	*150.9* ± *255.4*	*560.0* ± *487.5*	*254.4* ± *375.6*
LEDD (mg)	*618.6* ± *532.6*	*471.0* ± *501.0*	*271.3* ± *304.3*	*185.4* ± *298.6*	*625.0* ± *553.8*	*330.0* ± *493.2*
↑ CSFp (%)	*25.9*	*15.4*	*17.6*	*35.3*	*12.5*	*18.2*
↑ CSFcc (%)	*0.0*	*0.0*	*0.0*	*5.9*	*0.0*	*0.0*
Motor complaints (%)	*28.4*	*16.4*	*82.6*	*62.1*	*70.0*	*55.6*
Sensory complaints (%)	*49.0*	*30.9*	*39.1*	*37.9*	*60.0*	*61.1*

Data are presented as mean ± SD

*COMT* catechol-O-methyltransferase inhibitor, *CSFp* Cerebrospinal Fluid protein, *CSFcc* Cerebrospinal Fluid cell count, *HY* Hoehn and Yahr Stage, *MDS-UPDRS III* Movement Disorder Society Unified Parkinson’s Disease Rating Scale Part III, *MSA* multiple-system atrophy, *LDD* Levodopa Daily Dose, *LEDD* Levodopa equivalent daily dose, *MoCA* Montreal Cognitive Assessment, *PD* Parkinson’s Disease, *PNP* Polyneuropathy, *PSP* Progressive supranuclear palsy

^*^p < 0.05 compared with patients with and without PNP in each disease group

Among PSP patients, significantly more men were suffering from PNP than women (p = 0.039) and PSP patients with PNP were also significantly older than those without (72.7 ± 7.5 vs 66.9 ± 7.5, p = 0.0084). Despite a comparable disease duration (p = 0.35), patients with PSP demonstrated a significantly higher Hoehn and Yahr stage (p = 0.028) and MDS-UPDRS III scores (p = 0.0282), indicating more severe motor impairments.

None of the previously mentioned differences were detected between MSA patients with and without PNP.

### Electrophysiological and clinical characteristics of the PNP in PD, PSP, and MSA

**Fig. 1:**
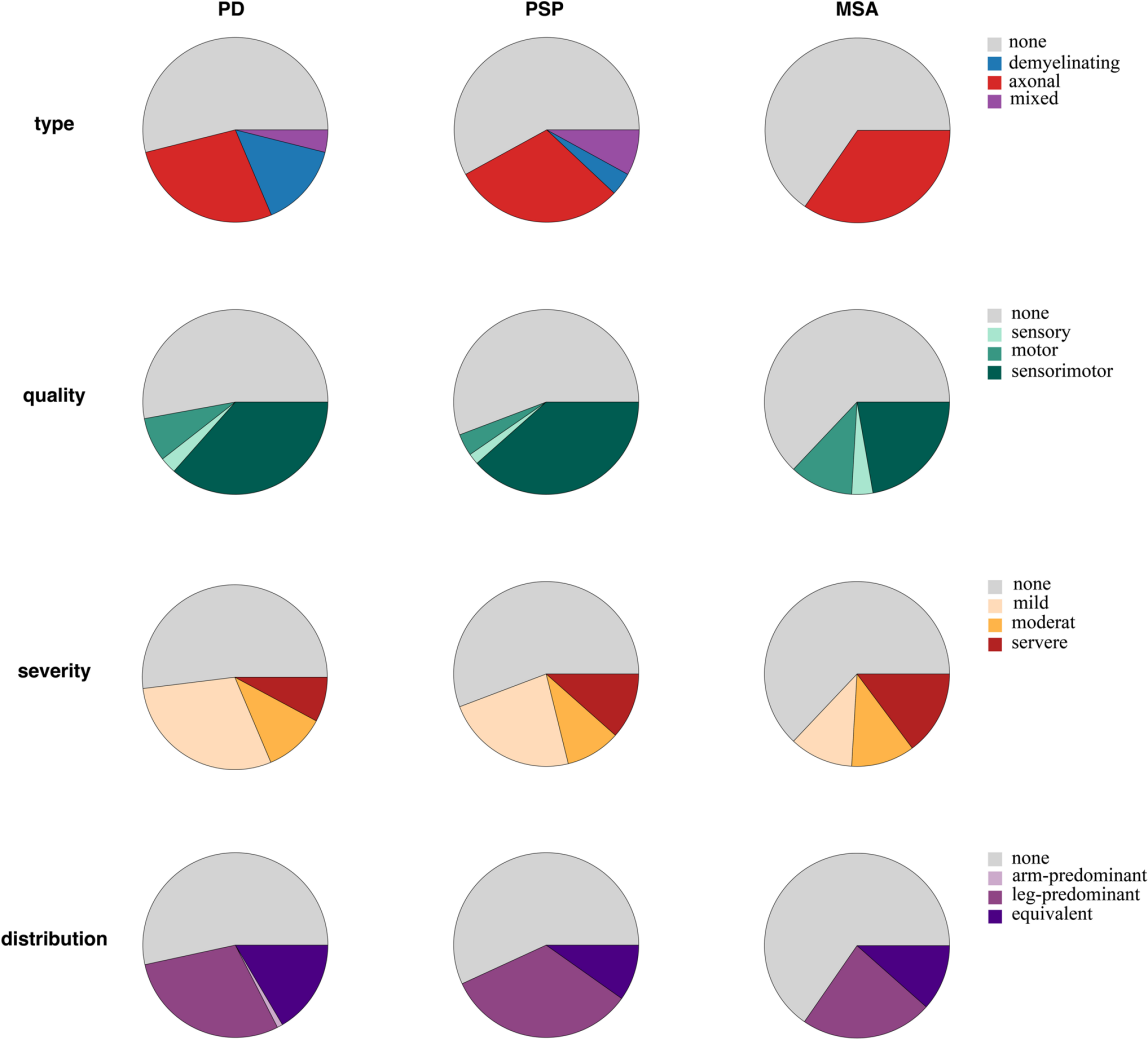
Characteristics of PNP in PD, PSP and MSA. It shows the type, location, quality and severity of the damage of PNP in PD, PSP and MSA. *MSA* multiple system atrophy, *PD* Parkinson’s Disease, *PSP* Progressive supranuclear palsy

The patients suffered mostly from an axonal-sensorimotor length-dependent polyneuropathy. There was no significant difference between PD, PSP, and MSA patients in any of the four PNP criteria. In each of the three disease groups, approximately half of the patients with PNP exhibited moderate or severe forms. Notably, over half of the MSA patients experienced a more severe form (Fig. 1).

**Fig. 2:**
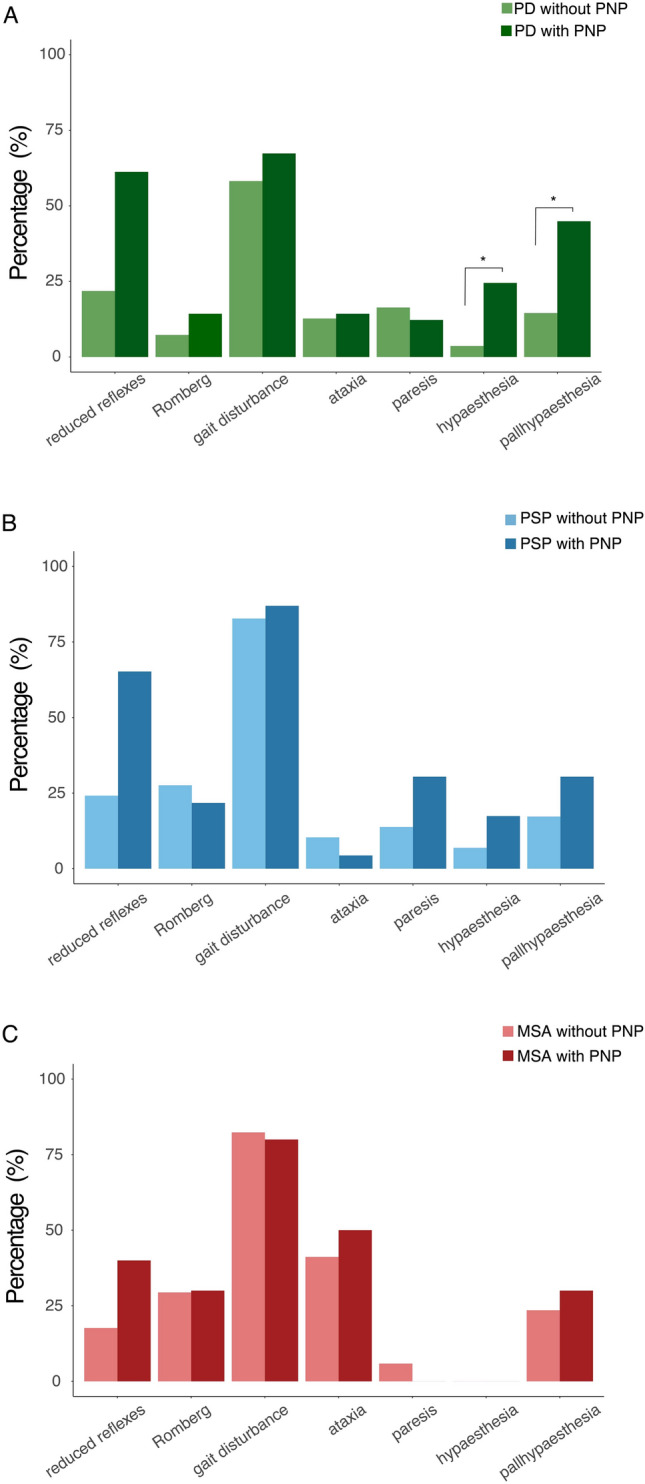
Distribution of clinical deficits in PD, MSA and PSP patients with and without PNP. This figure illustrates the prevalence of various clinical deficits in PD (**a**), PSP (**b**) and MSA (**c**) patients with and without PNP. The prevalence of signs and symptoms is compared within each disease group. *p < 0.05, Chi-squared test. *MSA* multiple-system atrophy, *PD* Parkinson’s Disease, *PNP* Polyneuropathy, *PSP* Progressive supranuclear palsy

Clinical complaints and neurological signs of PNP among PD, PSP, and MSA patients are shown in Fig. 2. ‘Gait disturbance’ was the most common and ‘reduced reflexes’ the second most common clinical deficit across patients with PNP in all the three disease groups. ‘Gait disturbance’ was as common in patients without PNP as in those with PNP (PD: p = 0.98, PSP: p = 0.56, MSA: p = 0.16). For MSA patients specifically, there was no significant difference between patients with and without PNP for any of the other mentioned symptoms. In PD and PSP patients with PNP, reduced reflexes were significantly more common than in patients without PNP (PSP: p = 0.041, PD: p = 0.008), while no difference in reflexes was found in MSA patients. PD patients with PNP also suffered significantly more often from hypesthesia (p = 0.021) and pallhypesthesia (p = 0.012) compared to those without PNP. Across all the three disease groups, approximately a quarter of patients had reduced reflexes and a small number of patients suffered from ‘pallhypesthesia’ despite the fact that PNP was not evident electrophysiologically. A similar distribution was also observed for ‘hypesthesia’ in patients with PD or PSP.

### Possible etiology of the PNP in PD, PSP, and MSA

**Fig. 3:**
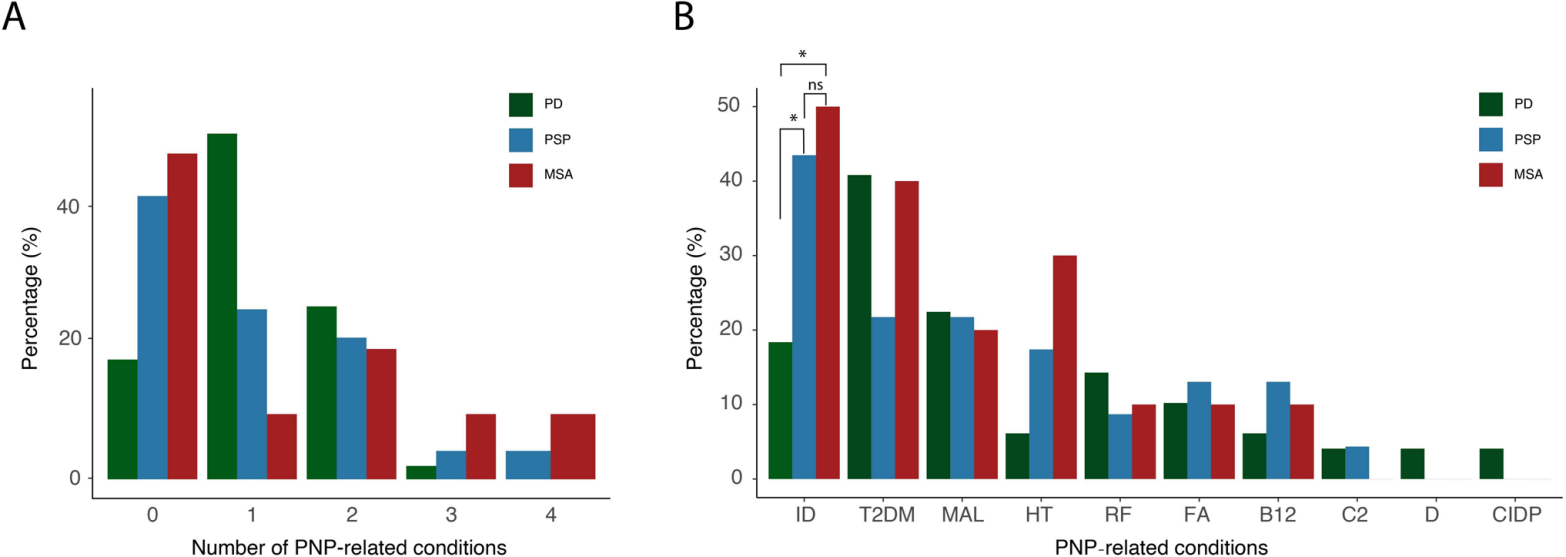
Distribution of PNP related conditions in PD, PSP and MSA patients. **a** The percentage of patients categorized by the corresponding number of PNP related conditions. **b** The percentage of each specific PNP related condition of PNP in PD, PSP and MSA patients, respectively. Pairwise Chi-Square tests were performed if the general Chi-Square test indicated significance (p < 0.05). *ns* not significant, *p < 0.05. *B12*: Vitamin B12, *C2*: Alcohol abuse, *CIDP* chronic inflammatory demyelinating polyradiculoneuropathy, *D* Drugs, *FA* Folic Acid, *HT* Hypothyreosis, *ID* Idiopathic, *MAL* malignancy, *RF* Rheumatoid factor, *T2DM* diabetes mellitus type 2

No specific etiology for PNP was detected in 18.4% of PD, 43.5% of PSP, and 50% of MSA patients with PNP (Fig. 3a). Patients without any specific cause were classified as ‘idiopathic’. This was the most common classification for both PSP and MSA patients. More than two PNP related diseases were identified in a small subset of PD, PSP, and MSA patients. T2DM was the most common cause for PNP among PD patients, and the second most common cause for PNP among PSP and MSA patients. Folic acid and vitamin B12 deficiencies were found in only a small number of PD, PSP, and MSA patients. Alcoholic polyneuropathy, drug-induced polyneuropathy, and CIDP were quite rare across all the three disease groups (Fig. 3b). No differences in the prevalence of T2DM as well as the other previously mentioned potential causes for PNP were detected between the patients with and without PNP across all the three disease groups (data not shown).

## Discussion

The present study is, to our best knowledge, one of few to comprehensively investigate and compare the prevalence, etiology, and influence of PNP in patients with PD, PSP and MSA in a large cohort. The results of this study showed that PNP is highly prevalent, not only among patients with PD, but also among patients with PSP and MSA. Furthermore, PNP among patients of all three types of Parkinsonism is mostly a sensorimotor axonal length-dependent polyneuropathy. Comorbidities such as TD2M and vitamin B12 deficiency, which are known common causes of PNP, were also present among the patients with Parkinsonism and T2DM was especially frequent. Additionally, we found no evidence that PD drugs had any effect on PNP.

Upon analyzing the specific clinical deficits associated with PNP, according to the clinical examination, PNP primarily resulted in reduced reflexes, hypesthesia and pallhypesthesia, although patients with PNP tended to describe more motor complaints in their self-reported symptoms compared to those without PNP. On the contrary, the electrophysiological abnormalities in our patients were mostly sensorimotor damages. It is plausible that, for example, gait disturbances from PNP were masked by those resulting from Parkinsonism, although the presence of PNP might have contributed to an exacerbation of the overall severity of gait disturbances. Additionally, PNP related gait disturbance is known to be rather sensory-related, which is consistent with our results, as there was a significantly higher prevalence of paresthesia/hypesthesia (Jahn et al. 2010) in PD patients. These findings support that PNP causes not only sensory deficits but also motor impairments.

Among PD and PSP patients, significantly more men suffered from PNP compared to women, suggesting that sex might be a determining factor for the pathogenesis of PNP. This is supported by prior findings (Hanewinckel et al. 2016b; Gierthmühlen et al. 2022; Visser et al. 2015). However, a study conducted in the general population showed opposite results (Hanewinckel et al. 2016a). This discrepancy may be due to differences between the general population and the older patients with Parkinsonism. Additionally, in both the PD and PSP group, patients with PNP were older than those without PNP. The current scientific evidence shows that patients of older age commonly show impaired nerve function in nerve conduction studies (Kleinveld et al. 2024) and the incidence of actual PNP increases (Hanewinckel et al. 2016b). However, while age might be a risk factor for the development of PNP, our study revealed a prevalence of 47% in PD patients, consistent with previous reports (Toth et al. 2010; Merola et al. 2017). Furthermore, patients with APS showed a significantly higher prevalence of PNP compared to the reported prevalence in the general population of a similar age. Therefore, age alone cannot explain the high prevalence of PNP in patients with Parkinsonism.

T2DM is a well-known and common cause for PNP. In our study, T2DM had also the highest prevalence among PNP-related conditions in PD patients with PNP and was the second most common cause for PNP in PSP and MSA patients after an idiopathic PNP. This is consistent with previous reports that investigated the association between T2DM and PD (Merola et al. 2017; Stockmann et al. 2025). In addition to T2DM, Vitamin B12 deficiency, folic acid deficiency, and alcohol abuse are also common causes for axonal PNP (Hanewinckel et al. 2016a; Sahin et al. 2010; McCombe and McLeod 1984), which is consistent with the type of the damage in our patients. However, all these comorbidities had a low prevalence in the studied cohort. Moreover, we cannot exclude the possibility that patients were supplementing over-the-counter B vitamins without prescriptions, as the importance of addressing vitamin deficiencies has been emphasized. Furthermore, some patients might not admit their history of elevated alcohol consumption. However, the similar prevalence of these conditions between patients with and without PNP makes it difficult to establish them as the primary etiology of PNP in individuals with Parkinsonism.

Levodopa has been considered to play a causative role in the development of PNP in PD (Romagnolo et al. 2019; Cossu et al. 2016; Loens et al. 2017). The impact of levodopa therapy on PNP risk was shown to be more pronounced with levodopa/carbidopa intestinal gel infusion (Romagnolo et al. 2019). In our study, we did not find negative, PNP-related effects of levodopa in any of the three groups with Parkinsonism. These findings suggest that no special concern about negative effects of levodopa on neuropathies is needed when treating such patients. Moreover, it was reported that COMT inhibitors could have a protective effect on restoring these metabolic imbalances and thus preventing the development of PNP in PD patients (Cossu et al. 2016). In our study, we did not find any indication for such protective effects of COMT inhibitors. Larger studies are necessary to further evaluate this point.

In the general population, around 25% of PNP cases are idiopathic (Hanewinckel et al. 2016a). In our study, idiopathic etiology was the second most common cause of PNP in PD patients with a prevalence of approximately 20%, following T2DM. However, in PSP patients, idiopathic neuropathy was more prevalent, affecting over 40% of the group. Notably, an even higher occurrence of this was found in MSA patients, with almost 50% of PNPs in this condition considered as idiopathic. A plausible explanation could be the emergence of abnormal proteinaceous aggregates in the peripheral nervous system, similar to those observed in the central nervous system among patients with neurodegeneration (Jucker and Walker 2018). PD is neuropathologically associated with the deposition of aggregated α-synuclein in neurons (Hayes 2019), while PSP is associated with a characteristic four-repeat tau neuropathology (Boxer et al. 2017), whereas MSA is mainly characterized by misfolded α-synuclein in oligodendroglial cytoplasmic inclusions (Poewe et al. 2022). Increasing evidence indicates that such abnormal proteinaceous aggregates are also expressed in various peripheral organs. α-synuclein deposits were not only detected in the gastroduodenal tract and skin of PD patients (Emmi et al. 2024; Gibbons et al. 2024) but also in the skin of MSA patients (Gibbons et al. 2024, 2023). Regarding this hypothesis, we assume that α-synuclein could also be present in the peripheral autonomic nerves of these patients and lead to autonomic deficits.

Moreover, both PD and PSP patients with PNP exhibited more severe Parkinsonism-related motor symptoms, despite having a similar disease duration. On one hand, we cannot exclude the possibility that PNP influences the course of the neurodegenerative disease, on the other hand, it is well known that the amount of abnormal proteinaceous aggregates in the brain is positively associated with the propagation of neurodegeneration (Jucker and Walker 2018). Thus, it is possible that there is a more severe involvement of such aggregates not only in both the central but also in the peripheral nervous systems of these patients. Further studies, including nerve biopsies, might be helpful to address this possibility. We did not find the same results for MSA patients. However, the small sample size of this group in the study reduces its external validity. Further studies with larger patient groups are necessary to deepen our understanding regarding this matter.

### Limitations

The lack of a control group including participants without Parkinsonism or the general population is a limitation of our study due to the nature of the university clinic setting. However, our focus is on comparing the prevalence of PNP between IPS and APS. Additionally, the comparison across the three cohorts but within the MSA group was limited due to the small size of the MSA group. Additionally, about 5.5% of the patients did not undergo the complete diagnostic PNP program at our hospital. For example, some tests were omitted because they had already been performed elsewhere, or some patients preferred for a shorter inpatient stay, completing further examinations as outpatients. The criteria we applied for assessing the severity of PNP do not account for age and body height and may be only suited to axonal PNP. Unfortunately, standardized criteria for the severity of PNP are not yet available. Some etiologies for polyneuropathy were not comprehensively documented or investigated during the acquisition of the data, such as alcohol abuse or genetic reasons. Furthermore, ENG only detects large-fiber neuropathies, and does not identify small-fiber neuropathies. Nevertheless, none of the patients in our cohort received a skin biopsy despite a small number of patients suffering from paresthesia and hypesthesia without electrophysiological evidence of nerve damage.

Overall, we think that further prospective studies with standardized and thorough assessments for different types of neuropathies would be important, especially for atypical Parkinsonian syndromes with small patient groups, such as MSA.

## Conclusion

In summary, PNP leads to not only sensory but also to motor impairments in patients with Parkinsonism. Because of the high prevalence, patients with Parkinsonism, especially the male and older patients, should receive a thorough screening for PNP and PNP related potential risk factors. Although conditions commonly associated with PNP, such as T2DM, vitamin B12 and folic acid deficiency, may not be the primary contributors to PNP in patients with Parkinsonism, it remains essential to screen for these conditions to prevent the negative effects on the progression of motor impairments. In the present study, no negative effects of levodopa on PNP were detected. The results suggest potential shared neurodegenerative pathways with abnormal proteinaceous aggregates. Further (biopsy) studies are needed to prove this hypothesis.

## Data Availability

The data supporting the findings of this study are available from thecorresponding author upon reasonable request.
